# Friend turned foe: selfish behavior of a spontaneously arising mitochondrial deletion in an experimentally evolved *Caenorhabditis elegans* population

**DOI:** 10.1093/g3journal/jkae018

**Published:** 2024-01-23

**Authors:** Abigail N Sequeira, Ian P O’Keefe, Vaishali Katju, Ulfar Bergthorsson

**Affiliations:** Department of Veterinary Integrative Biosciences, Texas A&M University, 402 Raymond Stotzer Parkway, College Station, TX 77845, USA; Department of Biology, Pennsylvania State University, 208 Mueller Laboratory, University Park, PA 16802, USA; Department of Veterinary Integrative Biosciences, Texas A&M University, 402 Raymond Stotzer Parkway, College Station, TX 77845, USA; Department of Biochemistry and Molecular Biology, University of Maryland, 655 W. Baltimore Street, Baltimore, MD 21201, USA; Department of Veterinary Integrative Biosciences, Texas A&M University, 402 Raymond Stotzer Parkway, College Station, TX 77845, USA; Program in Evolutionary Biology, Department of Ecology and Genetics (IEG), Evolutionsbiologiskt centrum, Norbyvägen 18D, Uppsala University, 752 36 Uppsala, Sweden; Department of Veterinary Integrative Biosciences, Texas A&M University, 402 Raymond Stotzer Parkway, College Station, TX 77845, USA; Program in Evolutionary Biology, Department of Ecology and Genetics (IEG), Evolutionsbiologiskt centrum, Norbyvägen 18D, Uppsala University, 752 36 Uppsala, Sweden

**Keywords:** selfish mitochondria, *Caenorhabditis elegans*, mutation, heteroplasmy, fitness, genetic conflict, mtDNA

## Abstract

Selfish mitochondrial DNA (mtDNA) mutations are variants that can proliferate within cells and enjoy a replication or transmission bias without fitness benefits for the host. mtDNA deletions in *Caenorhabditis elegans* can reach high heteroplasmic frequencies despite significantly reducing fitness, illustrating how new mtDNA variants can give rise to genetic conflict between different levels of selection and between the nuclear and mitochondrial genomes. During a mutation accumulation experiment in *C. elegans*, a 1,034-bp deletion originated spontaneously and reached an 81.7% frequency within an experimental evolution line. This heteroplasmic mtDNA deletion, designated as *meuDf1*, eliminated portions of 2 protein-coding genes (*coxIII* and *nd4*) and tRNA-*thr* in entirety. mtDNA copy number in *meuDf1* heteroplasmic individuals was 35% higher than in individuals with wild-type mitochondria. After backcrossing into a common genetic background, the *meuDf1* mitotype was associated with reduction in several fitness traits and independent competition experiments found a 40% reduction in composite fitness. Experiments that relaxed individual selection by single individual bottlenecks demonstrated that the deletion-bearing mtDNA possessed a strong transmission bias, thereby qualifying it as a novel selfish mitotype.

## Introduction

Natural selection on mitochondria operates at different levels of organization where competition takes place between populations, individuals, cells, and molecules of mitochondrial DNA within cells (Rand 2001). Depending on the species, cell types, and stages of development, cells can harbor between 1 to hundreds of thousands of mitochondria, and each mitochondrion can contain multiple copies of the mitochondrial genome. Continuously occurring mtDNA mutations result in heteroplasmy where novel mtDNA haplotypes are subject to genetic drift and natural selection both within and between individuals (Harrison *et al.* 1985; Kmiec *et al.* 2006; Stewart and Chinnery 2015; Karavaeva *et al.* 2017). One consequence of the hierarchical population structure of mtDNA is that selection can favor fast-replicating mitotypes within cells that are nonetheless detrimental to the individual (Eberhard 1980; Cosmides and Tooby 1981; Barr *et al.* 2005). Mitotypes that enjoy a replicative or transmission advantage, sometimes bypassing or exploiting the mitochondrial quality control mechanisms of the cell, have been referred to as “selfish” as their proliferation is at the expense of individual fitness.

Intracellular selection on mtDNA can be hampered by the lack of a direct relationship between the mtDNA genotype and phenotype. In a heteroplasmic cell containing wild-type (WT henceforth) and mutant mtDNA, the consequences of reduced mitochondrial function and cellular respiration caused by mtDNA mutations may affect all mtDNA molecules, regardless of whether they contain the mutant mtDNA or not. Similarly, beneficial mtDNA mutations improving mitochondrial function may extend their fitness benefits to all mtDNA genomes in the cell. Furthermore, cells frequently respond to detrimental mtDNA mutations by increasing mtDNA copy number, a short-term solution that contributes to the proliferation of deleterious mitotypes within cells. These scenarios, referred to as the “tragedy of the cytoplasmic commons,” could lead to unchecked proliferation of deleterious mitochondrial mutations within cells with severe negative fitness consequences at the individual levels (Haig 2016). Indeed, mtDNA heteroplasmy has been implicated in mitochondrial diseases associated with senescence, infertility, and cancer in animals (Zeviani and Antozzi 1997; Wallace 1999; Keogh and Chinnery 2013; Wallace and Chalkia 2013; Greaves *et al.* 2014; Stewart and Chinnery 2015; Lakshmanan *et al.* 2018). Nuclear genomes have responded to these potentially catastrophic situations with their own adaptations, which include prevention (e.g. mtDNA repair), increasing the efficiency of selection on mtDNA variation within individuals, and compensatory mutations that ameliorate the effects of deleterious mtDNA mutations (Haig 2016; Havird *et al.* 2019). Small mtDNA genome size, uniparental inheritance, mtDNA copy number bottlenecks during oogenesis, mitophagy, and apoptosis contribute to mtDNA quality control in animals (Haig 2016; Havird *et al.* 2019). Studying how certain mtDNA genotypes evade these mechanisms that maintain mitochondrial health and prevent the proliferation of detrimental mtDNA mutations helps us understand the coevolutionary processes of mitonuclear interactions (Estes *et al.* 2023). To this end, we also need to discern the types of mutations and their repertoire of selfish properties that nuclear-encoded mtDNA quality control mechanisms evolved to suppress.

*Caenorhabditis* nematodes have emerged as an important model for studying mitochondrial biology and evolution with implications for ageing, disease, and evolution (Estes *et al.* 2023; Onraet and Zuryn 2024). Several mtDNA deletions in *Caenorhabditis* display selfish properties in that they are strongly detrimental and, yet, persist over long periods in experimental populations and can reach high intraindividual frequency (Tsang and Lemire 2002a; Liau *et al.* 2007; Estes *et al.* 2011; Clark *et al.* 2012; Konrad *et al.* 2017; Ahier *et al.* 2018; Dubie *et al.* 2020). The first documented and most studied case of a selfish mitochondrial deletion in *Caenorhabditis elegans* is *uaDf5*, which is lacking close to 25% (11 genes) of the mitochondrial genome, including *atp6*, *ctb-1*, *nd1*, and *nd2* (Tsang and Lemire 2002a). The *uaDf5* deletion has persisted in laboratory populations in 60% frequency despite significant negative consequences for fitness-related traits. Additional heteroplasmic mtDNA deletions in *C. elegans* with long-term stability in laboratory populations include a 179-bp deletion in *nd1*, a 1-kb deletion spanning *atp6*, *nd2*, and 3 tRNA genes, and a 4.2-kb deletion spanning *coxI*, *coxII*, *nd5*, *16rRNA*, and 5 tRNA genes (Gitschlag *et al.* 2016; Meshnik *et al.* 2022). Collectively, these 4 mtDNA deletions include elimination of DNA from 8 out of 12 protein-coding genes in the *C. elegans* mitochondrial genome. Furthermore, several natural isolates of the congeneric *Caenorhabditis briggsae* harbor selfish mtDNA deletions in *nd5* (*nad5Δ*; Estes *et al.* 2011; Clark *et al.* 2012).

During a mutation accumulation (MA henceforth) experiment with obligately outcrossing *C. elegans* experimental lines bearing a *fog-2* null mutation and subjected to RNAi-induced knockdown of a key mismatch repair gene *msh-2* (Katju *et al.* 2008, 2022), a spontaneous 1,034-bp deletion originated in 1 experimental line (line 16). This deletion, designated as *meuDf1*, spanned the 3′ end of *coxIII* and tRNA-*thr* and the 5′ end of *nd4* and had reached 81.7% frequency within this line at the termination of the MA experiment. Additionally, this heteroplasmic mtDNA deletion is linked to a nonsynonymous point mutation in *nd4L*, which was estimated to be at 93.7% frequency (Katju *et al.* 2022). Here, we analyze this mitotype's impact on key fitness-related traits, its population dynamics, and test for selfish behavior that may have facilitated its spread within this population.

## Materials and methods

### Sequestering the *meuDf1* mitochondria in a WT N2 background

The original MA experiment was performed in an obligately outcrossing strain caused by a loss-of-function mutation in the *fog-2* gene [*fog-2*(*lf*)] that knocks out the sperm production pathway in hermaphrodites, thereby converting them to obligately outcrossing females (Katju *et al.* 2008, 2022; Rane *et al.* 2010). To directly test the phenotypic effects of the mutant mitochondria without the confounding influence of other accumulated spontaneous nuclear mutations within this experimental MA line of *C. elegans*, the mtDNA of MA line 16 was sequestered in a WT N2 nuclear background. This was accomplished by successively backcrossing females carrying the heteroplasmic *meuDf1* mitotype to *fog-2*(*lf*) males with a WT (non-MA) nuclear background for 10 generations during which the contribution of the nuclear DNA of the MA line was reduced by half each generation. Finally, backcrossed females with the *meuDf1* mitotype were crossed with WT N2 males for another 3 generations (generations 11–13) to restore a functional *fog-2* allele and convert the females to functional hermaphrodites. The use of hermaphroditic lines enables ease of manipulation when performing experiments while enabling a clean assessment of fitness effects relative to other similar mutations that have been investigated in preceding studies (Dubie *et al.* 2020). These functional hermaphrodites with the *meuDf1* mitotype were retained to establish 3 replicate lines with the *meuDf1* mitotype from MA line 16 in a WT nuclear background. These 3 lines (A–C) were cryogenically preserved at −80°C. After 13 generations of backcrossing, the proportion of the nuclear DNA from the original MA line is estimated to be 0.5^13^ of the total nuclear DNA, or approximately 1.2 × 10^−4^, which virtually removes all potential nuclear mutations that may have arisen during the MA procedure. During each backcross generation, the parent worms were screened via PCR (forward primer: 5′-AGTACCAGTACACGAGTTGGG-3′, reverse primer: 5′-AGAAGGTGGTACACCCCTATTTG-3′) to confirm the presence of the *meuDf1* mitotype. The PCR products were run on a 1% agarose gel (250 mL 1× Tris-acetate EDTA; 1 g agarose; 1 *μ*L GelRed) at 105 V for 45 min. The expected band sizes were 490 bp and ∼1,500 bp for the mutant and WT band, respectively.

### Phenotypic assays for 4 fitness-related traits

Four fitness assays were performed on the 3 backcrossed lines bearing the *meuDf1* mitotype. The 4 life history traits tested were productivity, survivorship to adulthood (survivorship), longevity, and developmental rate. Prior to conducting the fitness assays, frozen stocks of N2 WT (control) and the backcrossed lines (A–C) were thawed, and a parental generation was established for all lines. Four days later, the F1 generation was established by setting up 20 replicates for 4 control N2 lines (*n* = 80) and 15 replicates for each of the 3 experimental lines (A–C; *n* = 45). This was done by placing a single L4 hermaphrodite on a 35-mm NGM agar plate seeded with *Escherichia coli*  OP50. To rid the worms of potential freezer effects, an L4 hermaphrodite was transferred for each replicate onto a new plate every 4 days for 3 generations. The F4 generation was used to conduct the assay.

For the survivorship to adulthood (or maturity) assay, 10 L1 larvae were sequestered onto a 35-mm NGM agar plate seeded with *E. coli*  OP50. This was done for each replicate. Forty-eight hours later, the number of worms that were L4 or older was considered to have survived to adulthood. To assess survivorship, the number of adult worms was divided by the total number of L1 larvae initially sequestered. Survivorship values can range between 0 and 1. In *C. elegans*, development from egg to egg-laying adult typically occurs in 3.5 days. In this assay, we allowed 4 days for the sequestered worms to develop from the first larval stage (L1) to at least the fourth larval stage (L4), thereby allowing ample time for slow-developing worms to be counted as having survived to adulthood.

To measure development rate, a single L1 larva was placed on a 35-mm NGM agar plate seeded with *E. coli*  OP50. For each line, 15 individuals were used. Thirty-six hours later, each worm was assessed every 2 h for 24 h or until the individual reached adulthood. A worm was deemed as having reached adulthood when the first egg was observed entering the uterus. After the first 24 h, any worms that had not reached adulthood were checked every 4 h. Development rate was calculated by taking the inverse of the time in hours starting from the L1 larval stage for a worm to reach adulthood.

To measure productivity, the worms from the developmental rate assay were used. Each worm was placed on a 35-mm NGM agar plate seeded with *E. coli*  OP50 and allowed to lay eggs. Every 24 h, the mother worm was transferred to a new plate. This was repeated each day for 8 consecutive days. After the mother worm was removed from each plate, the plate was kept in a 20°C incubator for 24 h followed by storage at 4°C. The plates were kept at 4°C for 3 weeks, after which the progeny were counted. Counting was facilitated by adding 200 *μ*L of 0.075% solution of toluidine blue to the plate, which stains the agar but not the worms.

The same worms used for the development rate and productivity assay were used for longevity. After the last day of productivity, the worm was moved to a new NGM agar plate seeded with *E. coli*  OP50 and checked each day until mortality. An individual was considered dead when the worm was no longer moving after being lightly prodded or there was no pharyngeal activity observed. Longevity was calculated as the number of days the worm was alive, from the L1 larval stage to death.

### Analyses of fitness data

For each of the 4 fitness assays, data analyses were conducted using the program R. The relative fitness values for each trait in the mutant lines were calculated according to the mean absolute fitness of the N2 control lines, which were normalized to 1. The fitness measurements were determined by dividing the mean fitness of each trait observed in the mutant lines relative to that of the WT control lines.

### Competition assays

In order to explore the fitness consequences of the *meuDf1* mitotype under competitive conditions, we conducted a competition experiment. Frozen stocks of 3 backcrossed lines bearing the *meuDf1* mtDNA in a WT nuclear background (A–C) and N2 WT lines were thawed. To remove freezer effects, individual worms were transferred to NGM agar seeded with *E. coli*  OP50 plates for 2 generations. The F3 generation was used to establish 12 populations. We established 6 competed populations (A1, A2, B1, B2, C1, and C2) that consisted of equal ratios of *meuDf1*-bearing:WT worms (50 *meuDf1* and 50 WT individuals) onto 100 mm NGM plates seeded with an *E. coli*  OP50 lawn (1 mL). We also established 6 noncompeted populations (NCA1, NCA2, NCB1, NCB2, NCC1, and NCC2) of 100 *meuDf1*-bearing individuals onto 100 mm NGM plates seeded with an *E. coli*  OP50 lawn (1 mL). Every 4 days, each population underwent a standard *C. elegans* bleaching protocol with a 30% bleach and 15% 5 M NaOH solution. The bleaching regimen allowed us to synchronize each generation by only transferring the eggs, thus excluding previous-generation adult worms from contributing to the gene pool. The competed populations were established every 4 days and maintained for 15 successive generations. The noncompeted populations were maintained for a total of 60 generations following the same bleaching protocol.

To determine the frequency of *meuDf1-*bearing individuals present in the 12 populations, 30 adult *C. elegans* were randomly selected each generation across the 15 generations of the competition experiment and subjected to a single worm lysis protocol to extract DNA. The single worm lysis product was used for genotyping via PCR. Three primers were used to determine the presence of the *meuDf1* mutation, 1 forward and 2 reverse, with the second reverse primer located within the deletion: 5′-AGTACCAGTACACGAGTTGGG-3′, 5′-AGAAGGTGGTACACCCCTATTTG-3′, and 5′-AATTCTAACAAAGCTACTAGAAACCTT-3′, respectively. Individuals bearing the mutation were expected to display both the mutant band (490 bp) and the WT band (350 bp) since *meuDf1*-bearing worms are heteroplasmic for the mutant mitotype. WT individuals were expected to display only the WT band (350 bp). The PCR products were run on a 1.5% agarose gel (250 mL 1× Tris-acetate EDTA; 3.75 g agarose; 1 *μ*L GelRed) at 105 V for 45 min.

The relative fitness, *w*, of worms harboring the *meuDf1* heteroplasmy was calculated from their average frequency across competition experiments comprising 6 replicate populations over 8 generations. The slope of the regression line for the log (*p*/*q*) in each generation *t* was calculated, where *p* is the average frequency of individuals harboring the *meuDf1* mitotype, and *q* equals the average frequency of individuals that only carry the WT mtDNA. The slope of the regression line equals log(*w*).

### Testing for replicative advantage of the *meuDf1* mitotype via an evolutionary replay experiment

Deleterious mtDNA mutations, such as the *meuDf1* mitotype can, in principle, reach high frequencies by (1) chance (genetic drift) or by (2) some form of replicative or transmission advantage within individuals. In order to determine whether the *meuDf1* mitotype possessed a replicate or transmission advantage, we conducted an evolutionary replay experiment. Replay experiments using experimental evolution approaches in the laboratory serve to investigate the roles of contingency and determinism in the repeatability of evolutionary outcomes (Blount *et al.* 2018). To test if the *meuDf1* mitotype has a competitive advantage relative to WT mtDNA within individuals, or reached a high frequency during MA due to genetic drift, we need to minimize the effects of competition between individuals. This can be accomplished by bottlenecking the *C. elegans* lines at a single individual in every generation. This design makes clear predictions regarding intraindividual selection vs genetic drift. If the frequency of *meuDf1* is determined by genetic drift (chance events), there should be no significant change in the average frequency of *meuDf1* across multiple independent lines that are descended from the same ancestor although the interline variation in the frequency of *meuDf1* is expected to increase with the number of generations. In contrast, if the *meuDf1* mitotype has a competitive advantage within individuals, the average *meuDf1* frequency will increase. Lastly, within-individual selection against *meuDf1* would result in decreased *meuDf1* frequency across the replicate lines.

As described above under the protocol for competition assays, 6 noncompeted populations heteroplasmic for *meuDf1* were maintained at high population density by chunk transfer for 60 generations, which resulted in selection against high within-individual frequency of the deletion. We screened 90 randomly selected hermaphrodites (15 random individuals from each of the 6 populations) to determine the *meuDf1* heteroplasmic frequency via digital droplet PCR (ddPCR) after they had laid eggs over a 2-day period. For ddPCR, the individuals were lysed and the single worm lysates diluted 1:50 with molecular-grade water. Two Bio-Rad ddPCR fluorescent probes (FAM and HEX) were used to determine the frequency of WT to *meuDf1* mtDNA in each sample. The FAM probe targeted a mtDNA segment inside the deletion and hence was used to estimate the concentration of WT *nd4*. The HEX probe targeted mtDNA outside the deletion in *nd1* and was used to estimate the concentration of total mtDNA (*meuDf1* + WT). Probe designs are proprietary but see Table 1 for the amplicon context of the probes. *meuDf1* mtDNA frequency was calculated by subtracting the ratio of FAM to HEX concentration from 1. Using this screening procedure, we identified a hermaphrodite with a 34% *meuDf1* frequency. The offspring of this individual were sequestered to establish 15 replicate lines as part of the evolutionary replay experiment. These 15 experimental evolutionary replay lines were bottlenecked and maintained via single worm transfer each generation for 10 consecutive generations. In generations 1, 5, and 10, the mothers (*n* = 15) were allowed to lay eggs over a 2-day period and then lysed to determine their *meuDf1* heteroplasmic frequency via ddPCR as described above.

**Table 1.** Description of ddPCR probes for Bio-Rad ddPCR assay for quantification of mtDNA copy number.

Gene	Probe fluorophore	Location	Amplicon context	Amplicon length
*nd4*	FAM	mtDNA: 7282..7404	TTTTTACATCTTTGATTACCTAAAGCTCATGTAGAGGCTCCTACA ACAGCTAGAATACTTTTAGCTG GATTACTATTAAAATTAGGCAC AGCGGGATTTTTACGTATTTTAGGTAGTTTAAGA	89 bp
*nd1*	HEX	mtDNA: 2096..2218	GTCATTTATTGGGAAGAAGACAAAATCGTCTAGGGCCCACCAAG GTTACATTTATGGGATTA GCACAAGCTTTATTGGATGGGGTTAAA CTTTT AAAAAAAGAACAAATAACACCCTTAAATT	97 bp

### Copy number analysis

Total mtDNA copy number was estimated using ddPCR. Randomly chosen 15 L4 *C. elegans* bearing the *meuDf1* heteroplasmy from each of the 6 backcrossed lines (NCA1, NCA2, NCB1, NCB2, NCC1, and NCC2), 90 individuals in total, were compared with 15 N2 WT individuals. A standard single worm lysis protocol was used to extract DNA from individual worms. We used a Bio-Rad FAM probe designed to target the single-copy nuclear gene, *daf-3*, and a Bio-Rad HEX probe targeting the *ctb-1* mitochondrial gene. The ratio of mitochondrial DNA to a single-copy nuclear gene was estimated by dividing the concentration of DNA bound to the HEX probe by the concentration of DNA bound to the FAM probe. Data were analyzed using R.

## Results

### A deletion spanning 2 protein-coding genes was detected at a high frequency

**Fig. 1.**
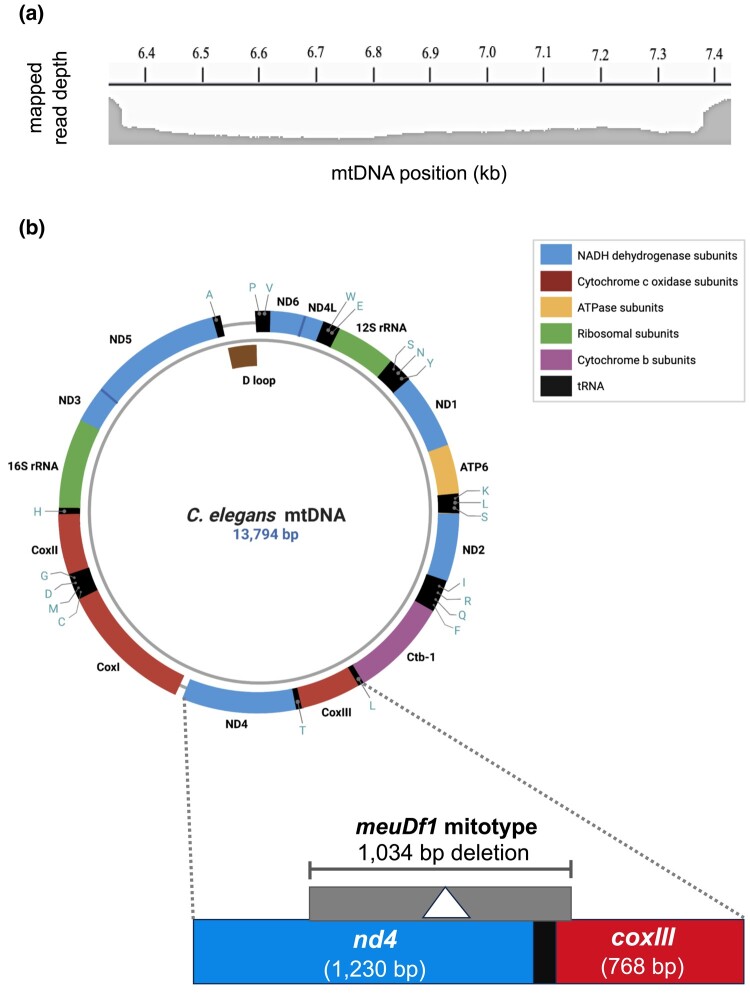
Identification and location of the *meuDf1* deletion in a MA line of *C. elegans*. a) A 1,034-bp frameshift mtDNA deletion spanning the *coxIII/tRNA-thr/nd4* genic region occurred spontaneously in a *C. elegans* MA line and was detected via WGS (Katju *et al.* 2022). We refer to this deletion as *meuDf1*. The figure displays the read depth from Illumina WGS mapped to this genic region of the *C. elegans* mitochondrial genome in line 16. The read depth inside the deletion is only 18.3% of the read depth of the sequences immediately flanking this deletion, thereby suggesting an 81.7% frequency of the *meuDf1* deletion within the experimentally evolved line. b) A map of the *C. elegans* mitochondrial genomes (adapted from Okimoto *et al.* 1992) displaying the genomic location, span, and the sequence context of *meuDf1*. Created with BioRender.com.

A 1,034-bp deletion starting near the 3′ end of *coxIII* and extending into *nd4* (Fig. 1a) arose spontaneously in a *fog-2 C. elegans* line subjected to repeated *msh-2* knockdown via RNAi during an MA experiment. The RNAi-induced knockdown of *msh-2*, a key mismatch repair gene, increased the nuclear mutation rate but was not found to directly affect the mtDNA mutation rate (Katju *et al.* 2022). Read depth analysis suggested 81.7% of the mtDNA in this particular MA line contained the deletion, which spanned 11.4% of *coxIII*, all of the intervening *tRNA-thr*, and 68.2% of *nd4* (Fig. 1b). In addition to this deletion designated as *meuDf1*, the mtDNA also contained a nonsynonymous base substitution (Leu → Pro) in the *nd4L* gene with an estimated heteroplasmic frequency of 93.7% based on whole-genome sequencing (WGS) analysis (Katju *et al.* 2022). Given the near fixation of the base substitution in *nd4L* and the high frequency of the deletion, we infer that the 2 mutations are linked. Furthermore, previous experiments in *C. elegans* have found no evidence for recombination between heteroplasmic mitochondrial deletions and base substitutions (Dubie *et al.* 2020). We refer to this deletion and nonsynonymous base substitution in this experimental line as the *meuDf1* mitotype.

### The *meuDf1* mitotype causes a significant fitness reduction

**Fig. 2.**
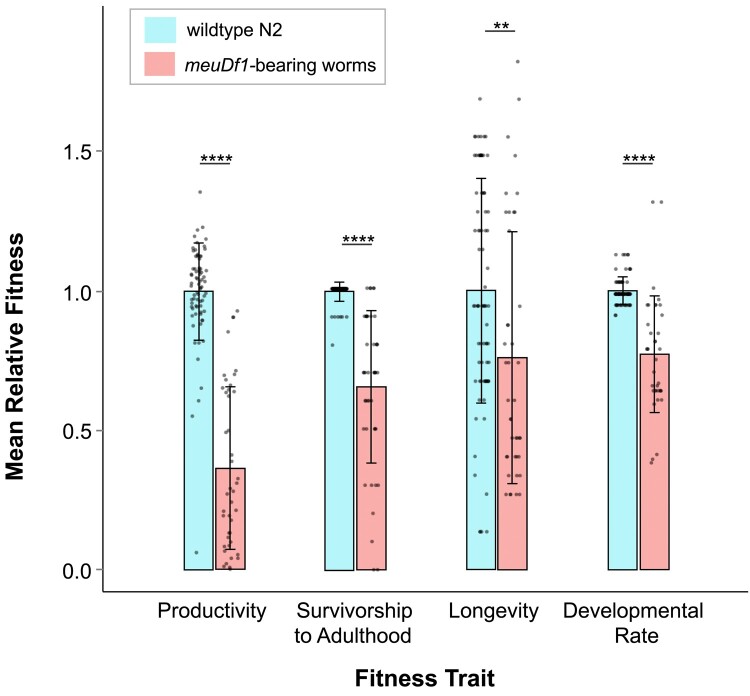
Relative trait means ± SD of the *meuDf1*-bearing replicate lines vs the WT N2 control. Phenotypic assays were conducted for 4 life history traits, namely (1) productivity, (2) survivorship to adulthood, (3) longevity, and (4) developmental rate. Mean fitness values for each of the 4 traits were measured across 3 backcrossed MA16 lines, each with 15 replicates where possible (*n* = 45) and 4 N2 control lines with 20 replicates each (*n* = 80). For simplicity, the mean relative fitness value for each of the 4 traits in the WT N2 control was scaled to a value of 1. There was a significant decrease in the mean trait value in all the *meuDf1*-bearing lines relative to the WT N2 lines. **P ≤ 0.01 and ****P ≤ 0.0001.

Following the backcrossing regime into a WT N2 genetic background, the average heteroplasmic frequency of the *meuDf1* deletion was determined to be 86.1%, which is similar to its estimated frequency from WGS analyses by Katju *et al.* (2022). The fitness effects of the *meuDf1* mitotype were tested in a WT Bristol N2 genetic background. The backcrossed lines containing the *meuDf1* mitotype were tested for 4 fitness traits: productivity, survivorship to adulthood, longevity, and developmental rate (Supplementary Table 1). These 4 traits were assayed in 3 independently backcrossed lines (A–C) harboring the *meuDf1* mitotype and 4 control lines with WT mitochondria. The relative fitness of the 3 mutant lines was significantly decreased in comparison to the WT controls (Table 2 and Fig. 2). The *meuDf1*-bearing lines had, on average, a 63.7% reduction in productivity compared to the WT control lines (Fig. 2; Wilcoxon rank sum, *z* = 8.43, *d.f.* = 1, *P* < 0.0001). The productivity of *meuDf1-*bearing lines ranged from 97–134 progeny over the course of 8 days relative to 19–418 for the WT controls. There was no significant difference in productivity between the 3 backcrossed deletion-bearing lines (Kruskal–Wallis rank sum, *χ*^2^ = 2.50, *d.f.* = 2, *P* = 0.29). The survivorship to adulthood of *meuDf1*-bearing lines was 34% lower than that of the WT control lines (Fig. 2; Wilcoxon rank sum, *z* = 9.12, *d.f.* = 1, *P* < 0.0001). On average, only 49% of L1 larvae survived to adulthood in line C, whereas survivorship in lines A and B ranged from 71 to 75%. Survivorship of the WT control lines ranged from 81 to 100%. With respect to survivorship, line C was significantly different from lines A and B, but A and B were not significantly different from each other (Kruskal–Wallis rank sum, *χ*^2^ = 9.52, *d.f*. = 2, *P* = 0.01). The average life span of *meuDf1*-bearing lines was significantly reduced relative to the WT controls (∼11 days vs 15 days, respectively; Fig. 2; Wilcoxon rank sum, *z* = 3.12, *d.f*. = 1, *P* = 0.0018). This corresponds to an approximately 27% decrease in longevity. There was no significant difference in longevity between the backcrossed *meuDf1*-bearing lines (Kruskal–Wallis rank sum, *χ*^2^ = 0.62, *d.f.* = 2, *P* = 0.73). Lastly, the *meuDf1-*bearing nematodes had significantly delayed development compared to the N2 control (Fig. 2; Wilcoxon rank sum, *z* = 7.07, *d.f*. = 1, *P* < 0.0001). The average time from egg to adult of *meuDf1*-bearing and WT worms was 66.3 and 47.6 h, respectively. This corresponds to the mutant-bearing worms taking, on average, 39% longer to reach adulthood. Conversely, the developmental rate of the *meuDf1*-bearing worms dropped by 28%. There was no significant difference in the rate of development between the 3 *meuDf1-*bearing backcrossed lines (Kruskal–Wallis rank sum, *χ*^2^ = 0.76, *d.f.* = 2, *P* = 0.69).

**Table 2.** Fitness of *meuDf1*-bearing mtDNA in a WT nuclear background relative to control worms of the Bristol N2 laboratory strain harboring WT mtDNA.

	Fitness-related trait			
	Productivity	Survivorship to adulthood	Longevity (days)	Developmental time (h)
${\bar{z}}_{N2\;\mathrm{control}}$	307.95	0.99	14.81	47.57
${\bar{Z}}_{meuDf1-\mathrm{bearingline}16}$	111.67	0.65	10.98	66.25
${\bar{z}}_{N2\;\mathrm{control}\;1}$	295.79	0.995	16.10	47.26
${\bar{z}}_{N2\;\mathrm{control}\;2}$	293.00	0.990	14.45	47.79
${\bar{z}}_{N2\;\mathrm{control}\;3}$	326.50	0.990	15.35	47.33
${\bar{z}}_{N2\;\mathrm{control}\;4}$	317.47	0.985	13.35	47.89
${\bar{Z}}_{meuDf1-\mathrm{bearingline}16.A}$	104.57	0.710	10.92	66.83
${\bar{Z}}_{meuDf1-\mathrm{bearingline}16.B}$	96.80	0.760	11.20	64.92
${\bar{Z}}_{meuDf1-\mathrm{bearingline}16.C}$	133.64	0.490	10.83	67.00

Twenty replicates each of 4 N2 control lines (*n* = 80) and 15 replicates each for 3 *meuDf1*-bearing lines (*n* = 45) were assayed. Estimates of the mean phenotype for 4 fitness traits are provided for the WT N2 control ( ${\bar{z}}_{N2\;\mathrm{control}}$; *n* = 80) and the *meuDf1*-bearing lines (${\bar{Z}}_{meuDf1-\mathrm{bearingline}16}$; *n* = 45). Mean fitness values across *n* = 20 and 15 replicates for individual control and experimental lines, respectively, are also provided (4 N2 control and 3 *meuDf1-*bearing lines).

### Severe fitness decrease in *meuDf1*-bearing worms in competition with WT

**Fig. 3.**
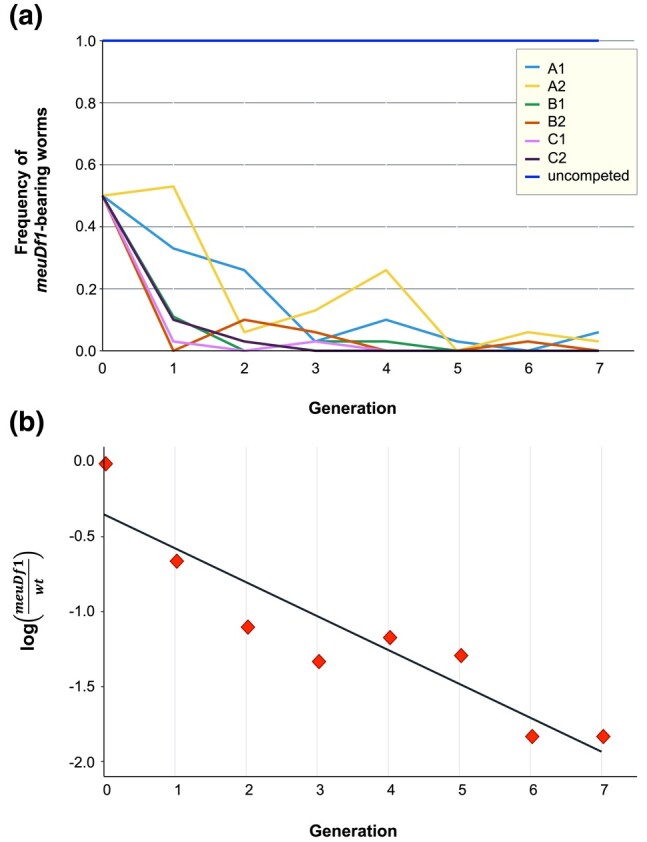
Evolutionary dynamics of *meuDf1* mtDNA under competitive conditions. a) Population-level frequencies of individuals harboring the *meuDf1* mtDNA heteroplasmy in 6 replicate lines under noncompeted vs competitive conditions. Each of the 6 populations in the competition experiment was established with equal ratios of *meuDf1* and WT mtDNA-bearing worms (50 *meuDf1*-bearing hermaphrodites in an N2 genetic background plus 50 WT mtDNA-bearing N2 hermaphrodites). The frequency of *meuDf1*-bearing individuals declines with time in competition with WT mtDNA. For comparison, the *meuDf1* heteroplasmy remained present under noncompetitive (control) conditions in every individual tested. b) A linear regression of the change in log[*f* (*meuDf1*/WT mtDNA)] with time (generations). The single data point for each generation represents the average for the 6 replicate populations. The relative fitness, *w*, of the *meuDf1* mutant mitotype was calculated from the slope of the regression line and estimated to be 0.59.

To explore the population dynamics of *meuDf1*, a competition assay was performed on the 3 backcrossed lines with 2 replicates per line (6 assays), wherein *meuDf1*-harboring worms were directly competed with worms containing WT mtDNA. There was a sharp decrease in the frequency of *meuDf1-*bearing worms in all 6 competition experiments (Fig. 3a). After 7 generations, individuals with the *meuDf1* mitotype were no longer detected in lines B1, B2, C1, and C2 and only persisted in low frequency in lines A1 and A2 (Fig. 3a). The average decline in frequency of the *meuDf1* mitotype was used to calculate its relative fitness (Fig. 3b). The slope of the linear regression for log(*meuDf1*/WT) was −0.23. The average relative fitness of worms carrying *meuDf1* was estimated at 10^−0.23^, which equals 0.59, corresponding to 41% lower fitness of *meuDf1*-bearing worms relative to WT. In addition, 2 replicates of each backcrossed *meuDf1* line were maintained for 60 generations at large population sizes as controls without competition with WT worms. In these noncompeted, large population size lines, the *meuDf1* mitotype was detectable in all worms tested after 60 generations. This suggests that the extinction or decrease of mutant-bearing worms in the competed populations was due to a competitive disadvantage and loss of *meuDf1*-harboring worms and not due to loss of the *meuDf1* heteroplasmy within worms (Fig. 3b).

### The *meuDf1* mitotype exhibits selfish drive

**Fig. 4.**
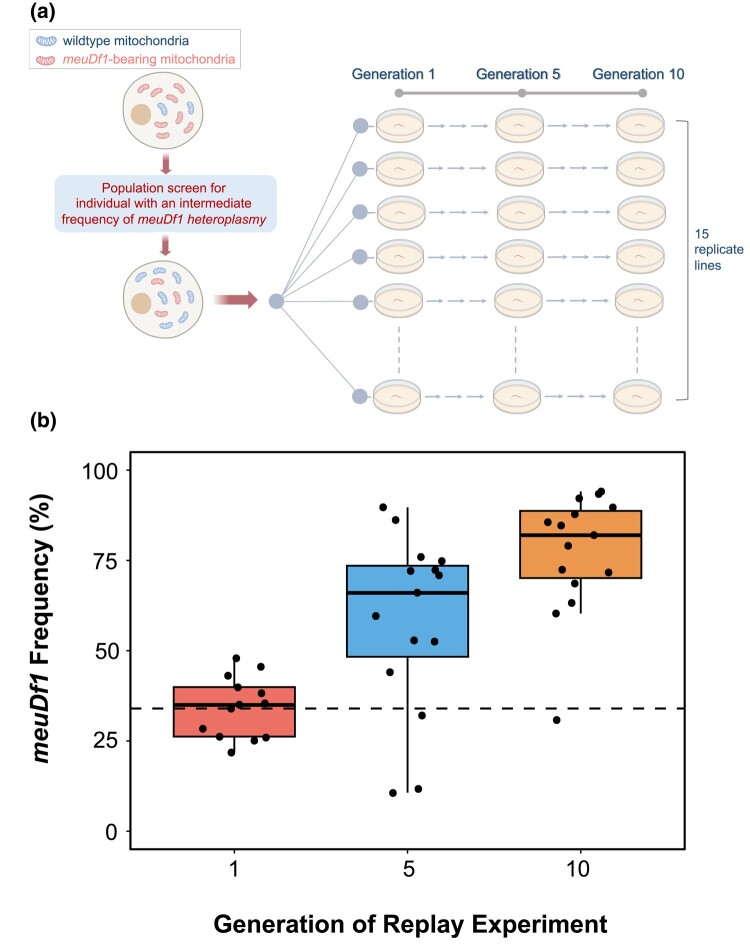
a) Schematic of the evolutionary replay experiment to test if the *meuDf1* mitotype has a competitive advantage relative to WT mtDNA within individuals. Ninety adult hermaphrodites were randomly selected from experimental populations maintained at large sizes and screened for *meuDf1* frequency via ddPCR (see *Materials and methods* for details). The offspring of a single worm with the *meuDf1* mitotype in 34% frequency were used to establish 15 experimental evolution lines, which were subjected to single individual bottlenecks each generation for 10 consecutive generations. The frequency of the *meuDf1* mitotype was measured by ddPCR in generations 1, 5, and 10. Created with BioRender.com. b) The change in heteroplasmic frequency of *meuDf1* during 10 generations of bottlenecking via single progeny descent in 15 replicate lines. The lines were established by 15 offspring of a single individual with 34% heteroplasmic frequency of *meuDf1*, indicated by a dashed line. The average heteroplasmic frequency across the 15 lines increased to 77%. There was a significant increase in the average frequency of *meuDf1* from generations 1 to 5 (34% to 56%, respectively; paired *t*-test: *t* = 5.82, *P* < 0.0001). Similarly, the average frequency of *meuDf1* increased significantly from generations 5 to 10 (56% to 77%, respectively; paired *t*-test: *t* = 6.06, *P* < 0.0001).

We conducted several experiments to test whether genetic drift or selfish drive contributed to the increased heteroplasmic frequency of *meuDf1* within this experimental *C. elegans* line. We used the offspring of an individual hermaphrodite harboring *meuDf1* at a 34% frequency as the parental generation to establish 15 replicate lines. In this follow-up experiment, we relaxed interindividual selection by subjecting these 15 replicate *meuDf1* lines to single individual bottlenecks in every generation for 10 consecutive generations as per an earlier protocol (Dubie *et al.* 2020). If the intraindividual dynamics are dominated by genetic drift, there should be no significant change in the average *meuDf1* frequency; however, the variance in heteroplasmy frequency between lines is expected to increase with time. If there is intraindividual selection against the *meuDf1* mitotype, the average frequency is expected to decline. Conversely, if the *meuDf1* has a replicative advantage, its average frequency is expected to increase. Over the course of 10 generations, the frequency of *meuDf1* more than doubled, rising to an average of 77% (Fig. 4; Supplementary Table 2). The average frequency of *meuDf1* in the first generation of the bottlenecked lines was similar to the parent line, with an average and median frequency of 0.34 and 0.35, respectively. These averages were calculated from ddPCR results of the single individuals used to initiate each of the 15 replicate lines. Although the individuals used to initiate replicate bottlenecked lines were siblings, the frequency of the deletion ranged from 0.22 to 0.48 and had a SD of 0.084. Between generations 1 and 5, the average frequency of *meuDf1* rose from 0.34 to 0.56 (paired *t*-test: *t* = 5.82, *P* < 0.0001). Between generations 5 and 10, the average *meuDf1* frequency rose further to 0.77 (paired *t*-test: *t* = 6.06, *P* < 0.0001). These results strongly suggest that the change in the frequency of *meuDf1* was not due to intracellular genetic drift but due to a replicative advantage (selfish drive) of the *meuDf1* mitotype.

### Copy number was significantly elevated in mutant lines

**Fig. 5.**
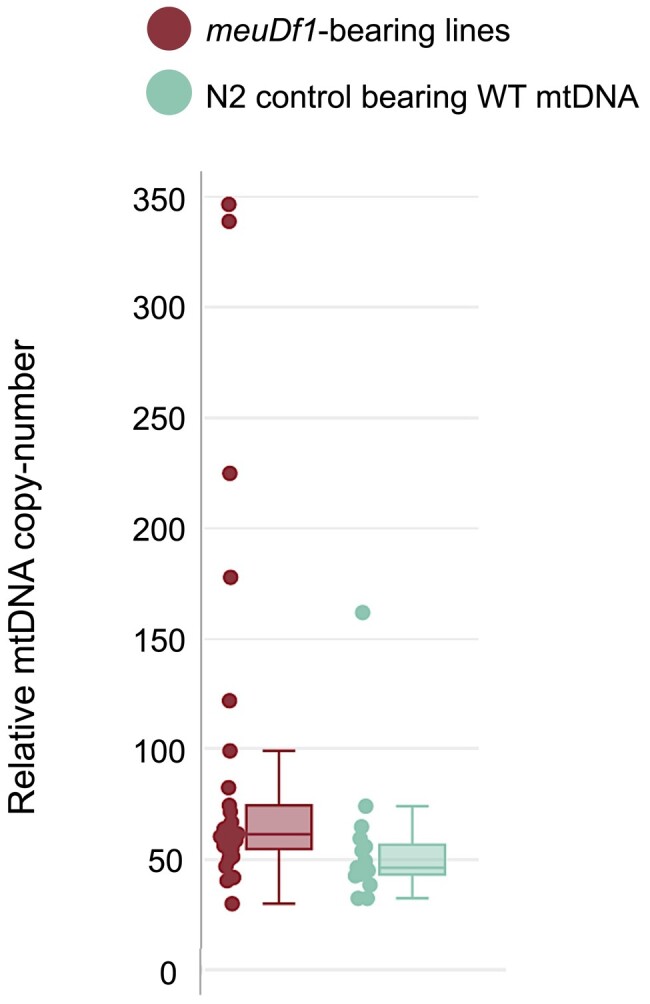
Relative mtDNA copy number in (1) 30 individuals of *meuDf1*-bearing lines and (2) 15 individuals of WT N2 control line as measured by ddPCR. The median relative mtDNA copy number in the *meuDf1* and WT mtDNA individuals was 62 and 46, respectively, thereby representing an increase of 32% in the former (Mann–Whitney test: *U* = 125, *P* = 0.004).

The relative copy number of mtDNA has frequently been observed to increase in the presence of a high-frequency deleterious mtDNA heteroplasmy (Tsang and Lemire 2002a; Gitschlag *et al.* 2016; Lin *et al.* 2016; Dubie *et al.* 2020). Furthermore, mtDNA copy number increase has been suggested as a driving force in the proliferation of deleterious mtDNA heteroplasmies (Tam *et al.* 2013; Gitschlag *et al.* 2016). The relative mtDNA copy number was estimated for 30 individuals with the *meuDf1* backcrossed into an N2 background and compared with mtDNA copy number in 15 N2 individuals with WT mtDNA (Fig. 5 and Supplementary Table 3). The median relative mtDNA copy number in the *meuDf1* and WT mtDNA individuals was 62 and 46, respectively, thereby representing an increase of 32% in the former (Mann–Whitney test: *U* = 125, *P* = 0.004).

## Discussion

The mitochondrial and nuclear genomes have been coevolving for approximately 1.5 billion years, resulting in the finely tuned expression of thousands of nuclear genes and several remnant mitochondrial genes that are essential for mitochondrial function (Lang *et al.* 1999). However, the high mutation rate of most animal mitochondrial genomes constantly creates heteroplasmy with the introduction of potentially harmful mtDNA mutations. Because the heredity of mtDNA is non-Mendelian, these harmful mtDNA mutations can sometimes proliferate if their deleterious fitness effects are limited to the sex that does not pass on mtDNA to future generations, or by a transmission or replication bias that favors the detrimental mutation (Eberhard 1980; Cosmides and Tooby 1981). Despite the evolution of a diversity of nuclear-encoded mechanisms to limit the proliferation of selfish or cheater mitochondrial genomes, harmful mtDNA mutations sometimes manage to reach high frequency under certain conditions (Haig 2016; Havird *et al.* 2019). These selfish mtDNA mutations provide us with an opportunity to probe the role played by genetic conflict in the evolution of the 2 respective genomes.

To qualify as a selfish mitochondrial mutation, a variant must fulfill 2 criteria: (1) it should have a neutral or deleterious fitness effect and (2) a transmission advantage. In the case of the *meuDf1* mitotype, we have herein demonstrated that the deleterious effects are large by any standard, including reduced productivity, a slower rate of development, lower survivorship to adulthood, and reduced longevity. Furthermore, composite fitness was reduced by 41% in individuals harboring this deletion when competed with individuals containing WT mtDNA. With such a large fitness disadvantage, a transmission advantage might be difficult to discern unless the efficiency of individual selection for reduced heteroplasmy frequency is limited to better reveal the within-individual dynamics. This was accomplished by single individual bottlenecks in every generation. Under these conditions, the average frequency of *meuDf1* increased rapidly in frequency from 0.34 to 0.77 in a mere 10 generations. The severe fitness cost combined with an increase in the frequency of the *meuDf1* mitotype when selection between individuals is limited serve as the hallmarks of a selfish mtDNA mitotype.

The identification of heteroplasmic mtDNA deletions in *Caenorhabditis* nematodes provide an opportunity to delineate their effects on organismal fitness and oxidative phosphorylation with their ensuing consequences for mtDNA copy number dynamics, mitonuclear coevolutionary interactions, and critical threshold ranges for the onset of disease phenotypes. As with any mutation, the fitness consequences of these heteroplasmic mtDNA deletions are likely to be context dependent and influenced by several factors including (1) the size of the deletion and, hence, the number of genes affected, (2) the mutational effect (frameshift vs in-frame), and (3) the heteroplasmic frequency, among others. However, this comes with the caveat that the influence of these factors on organismal fitness are confounded by differences in laboratory (rearing and environmental conditions), measures of different life history or fitness traits, and the use of different protocols. The *uaDf5* mtDNA deletion was identified in a laboratory population of *C. elegans* following an ethyl methanesulfonate (EMS) mutagenesis screen (Tsang and Lemire 2002b). This 3.04-kb deletion resulted in the partial or complete deletion of 4 protein-coding mtDNA genes (*nd1*, *atp6*, *nd2*, and *ctb-1*) and 7 tRNA genes and ranged in 20–80% frequency within laboratory lines with an average heteroplasmic frequency of ∼60% (Tsang and Lemire 2002b; Liau *et al.* 2007). A preceding study determined that the egg-laying frequency, longevity, and defecation frequency of ΔmtDNA-bearing worms were negatively correlated with *uaDf5* frequency (Liau *et al.* 2007). Our employment of a different set of key fitness traits and composite fitness inferred from competition experiments with *meuDf1*-bearing worms preclude a direct comparison. However, our measure of productivity, a key fitness trait that is extremely sensitive to mutation load and pressure (Katju *et al.* 2015, 2018) permits comparisons to the heteroplasmic Δ*nd5* deletion in wild isolates of *C. briggsae* (Estes *et al.* 2011) and the spontaneously arising Δ*ctb-1* deletion mitotype in another experimental evolution line of *C. elegans* (Dubie *et al.* 2020). The heteroplasmic Δ*nd5* deletion is unique in its occurrence in a natural setting in wild isolates of *C. briggsae*, a congeneric species of *C. elegans*. This in-frame deletion is expected to eliminate the first 262 aa (786 bp) of the WT protein product. Interestingly, *C. briggsae* wild isolates display considerable variation in Δ*nd5* frequency, ranging from 0–51.4% (Estes *et al.* 2011). Only 2 *C. briggsae* isolates (HK104 and HK105) with within-isolate Δ*nd5* frequencies ≥50% show a significant reduction in productivity, suggesting the presence of a “phenotypic threshold effect” wherein the presence of some proportion of WT mtDNA coexisting with mutated mtDNA confers a normal phenotype but results in an altered mutant phenotype once the mutant variant exceeds some critical (threshold) frequency (Rossignol *et al.* 2003). The *C. elegans* Δ*ctb-1* mitotype persists at a 96% frequency and results in a 499-bp frameshift deletion in the middle of the gene with a concomitant 65% decline in productivity (Dubie *et al.* 2020). Interestingly, *meuDf1* confers a similarly precipitous decline in productivity (∼64%) despite a lower heteroplasmic frequency of 82%. The greater length of the *meuDf1* frameshift deletion (1,034 bp) partially spanning 2 mtDNA genes (*nd4* and *coxIII*) may increase its potential for perturbation despite occurring at a lower frequency. It has been suggested that the critical threshold value is around 60% for mtDNA deletions (Shoffner *et al.* 1990; Rossignol *et al.* 2003; Chan 2006). While the Δ*ctb-1* and *meuDf1* heteroplasmies are clearly associated with significant productivity decline at high frequencies ≥80%, the Δ*nd5* case study (Estes *et al.* 2011) argues for the possibility of a lower phenotypic threshold effect depending on the focal gene(s) involved and other contextual characteristics (e.g. location and the potential to disrupt the reading frame).

The 2 protein-coding genes affected by deletion in the *meuDf1* mitotype are *coxIII* and *nd4* (Fig. 1). *nd4* codes for a hydrophobic inner subunit of ETC complex I and is involved in proton transfer (Hirst 2009; Efremov *et al.* 2010). *CoxIII* codes for one of the catalytic cores of ETC complex IV and is important for complex assembly (Capaldi 1990; Meunier and Taanman 2002). Studies have also confirmed that disruption to ETC complexes I and IV results in an increased production of free radicals, causing more oxidative stress on the mitochondria (Grad and Lemire 2004; Lenaz *et al.* 2004; Distelmaier *et al.* 2009). Hence, *meuDf1* likely leads to the misfolding of multiple subunits of the ETC complexes with the potential for engendering complex deficiencies. However, a determination of the direct impact(s) of the *meuDf1* mutation on mitochondrial function requires further study.

Mitochondrial DNA deletions have been associated with replication or transmission advantage in several systems, including *Saccharomyces*, *Drosophila*, and *Caenorhabditis* (Volz-Lingenhöhl *et al.* 1992; MacAlpine *et al.* 2001; Taylor *et al.* 2002; Tsang and Lemire 2002a, 2002b; Clark *et al.* 2012; Jasmin and Zeyl 2014; Dubie *et al.* 2020). Within *Caenorhabditis*, natural isolates of *C. briggsae* have been found with a deletion in *nd5* (*nad5Δ*), which increases in intracellular frequency when individual selection is relaxed, a classic symptom of selfish mtDNA and a conflict between different levels of selection (Clark *et al.* 2012). Furthermore, the transmission advantage of *nad5*Δ was frequency dependent within individuals, greatest when heteroplasmy levels of Δ*nad5* were low and diminishing with increased frequency of the deletion (Clark *et al.* 2012). The classic example of a selfish mtDNA deletion in *C. elegans* is *uaDf5*, which affects a quarter of the 12 protein-coding genes in the *C. elegans* mitochondrial genome, namely *atp6*, *ctb-1*, *nd1*, and *nd2* (Tsang and Lemire 2002a, 2002b; Liau *et al.* 2007). In addition, deletions that affect *atp6*, *coxI*, *coxII*, and *nd5* also have selfish properties in that they remain at high heteroplasmic levels in laboratory populations despite negative fitness effects (Gitschlag *et al.* 2016; Meshnik *et al.* 2022). Furthermore, these previously reported mtDNA deletions in *C. elegans* also span tRNA genes and, in one instance, a portion of a rRNA gene. Here, we show that a deletion spanning parts of 2 additional protein-coding genes and a tRNA also has a strong replication/transmission bias, bringing the total number of mitochondrially encoded protein genes in *C. elegans* that can be affected by selfish deletions to 10. The only mitochondrially encoded genes thus far that have not yet been reported as a part of a selfish mtDNA deletion in *C. elegans* are *nd6* and *nd4L*, which are just downstream of the major noncoding region thought to contain the replication origin. Furthermore, a second noncoding region located between *nd4* and *coxI* has also not been reported as exhibiting any deletions. Whether some deletions are “forbidden,” which might include mtDNA regions that are essential for replication and therefore cannot have selfish properties, remains to be seen.

The *meuDf1* heteroplasmy is associated with a significant increase in mtDNA copy number. An increase in total mtDNA copy number is commonly observed in organisms with severe mitochondrial mutations (Gitschlag *et al.* 2016, 2020; Filograna *et al.* 2019; Dubie *et al.* 2020). This is thought to be a compensatory mechanism aimed at sustaining mitochondrial oxidative phosphorylation, coordinated by the nuclear genome (Gitschlag *et al.* 2016). Ironically, this observed compensatory increase in mtDNA has been hypothesized to aid in the proliferation of mtDNA mutations (Tam *et al.* 2013; Gitschlag *et al.* 2016). It has been shown that ATFS-1, which accumulates in dysfunctional mitochondria, promotes the binding of the mtDNA polymerase POLG to mtDNA, which results in preferential replication of ΔmtDNA (Yang *et al.* 2022). Overreplication of mutant mtDNA in *C. elegans* should also apply to other classes of mtDNA mutations (base substitutions and small indels) resulting in nonsense mutations, frameshifts, and certain nonsynonymous mutations. However, there are no reported cases of base substitutions or frameshift mutations, which on their own have selfish mtDNA properties in *C. elegans*. This may stem from an ascertainment bias that favors easy to observe molecular changes such as large deletions or duplications. The deletion of critical genes is striking and demands an explanation whereas base substitutions might not elicit the same kind of attention. Some of the deletion heteroplasmies in *C. elegans* do contain additional base substitutions, which may contribute to their selfish properties. In the case reported here, these *meuDf1* molecules also harbor a nonsynonymous mutation in *nd4L*, and a previously reported mitochondrial *ctb-1* deletion is linked to mutations in *nd5* (Dubie *et al.* 2020).

The high mutation rate in mtDNA relative to the nucleus may predispose mitochondrial genomes to MA, and it is possible that a subset of deleterious mtDNA mutations are not just passively accumulating by genetic drift but are increasing in frequency by exploiting the nuclear regulation of mtDNA replication and bypassing mitochondrial quality control. The origin and proliferation of selfish organelles can lead to population extinction if left unabated but also sets the stage for compensatory evolution wherein nuclear-encoded changes may evolve to limit the spread of such renegade elements (Haig 2016). Furthermore, nuclear mutations may directly compensate for deleterious fitness consequences of mtDNA mutations, resulting in coadapted genes whose subsequent uncoupling may engender Bateson–Dobzhansky–Muller incompatibilities (BDMIs) (Sloan *et al.* 2017; Estes *et al.* 2023). Large-scale mtDNA deletions cannot reach fixation within individuals, and they are unlikely to survive in the wild considering their large fitness costs. Yet, a deletion in *nd5* has been found in natural isolates of *C. briggsae* (Howe and Denver 2008; Clark *et al.* 2012). However, these deletions provide us with an excellent opportunity to probe how deleterious mtDNA mutations can reach high frequency within individuals, the various adaptations to contain selfish mitochondria, and the evolutionary dynamics of mitonuclear genetic conflict.

## Supplementary Material

jkae018_Supplementary_Data

## Data Availability

All data including fitness data have been made available in the supplementary material file. The authors affirm that all data necessary for confirming the conclusions of the article are present within the article, figures, and tables. Supplemental material available at G3 online.
