# Supplementary material for: Friend turned foe: selfish behavior of a spontaneously arising mitochondrial deletion in an experimentally evolved Caenorhabditis elegans population
Source: G3 (Bethesda). 2024 Jan 23;14(4):jkae018. doi: 10.1093/g3journal/jkae018 (PMC11090458; doi:10.1093/g3journal/jkae018)
Supplement: jkae018_Supplementary_Data [file jkae018_supplementary_data.pdf]

Supplementary Material

Friend turned foe: selfish behavior of a spontaneously arising mitochondrial deletion in an experimentally evolved C. elegans population

Abigail N. Sequeira, Ian P. O'Keefe, Vaishali Katju, Ulfar Bergthorsson

G3: Genes | Genomes | Genetics

Supplementary Table S1. Fitness data for *meuDf1*-bearing worms and for *N2* control worms. The *meuDf1*-bearing worms consisted of three lines (A-C) each containing 15 mutant individuals and the control worms were 80 in total. Four life-history traits (survivorship, longevity, development time, and productivity) were assayed to determine the fitness of each individual. When available, data was collected for each trait. Grey cells indicate missing data.

Line	Replicate	Survivorship	Longevity (days)	Development Time (hrs)	Productivity
A	1	1	25	36	54
A	2	0.9	8	36	191
A	3	0			
A	4	0.8	8	120	30
A	5	0.8	5	72	26
A	6	0.6	6	72	59
A	7	1	7	54	95
A	8	0.3	11	115	2
A	9	0.8	12	67	20
A	10	0.9	19	47	151
A	11	0.8	6	49	83
A	12	0.5	7	71	59
A	13	0.6	9		214
A	14	1	19	63	195
A	15	0.7	22		285
B	16	0.7	4	50	89
B	17	1	12	50	278
B	18	0.9	7	50	262
B	19	0.6	4	74	6
B	20	0.7	11	78	40
B	21	0.9	27	80	119
B	22	0.9	13	56	40
B	23	0.9	4	56	16
B	24	0.8	20		153
B	25	0.5	6	74	203
B	26		13	74	0
B	27	1	19	74	3
B	28	0.7	7	50	196
B	29	0.7	7	78	12
B	30	0.3	14		35
C	31	0.5		52	278
C	32	0.5	18	58	198
C	33	0.3	4	60	65
C	34	0.7	6		12
C	35	0.7	5	50	126

C	36	0.9	23	58	209
C	37	0			
C	38	0.6	11	74	200
C	39	0.7	20	124	215
C	40	0.6	19	74	86
C	41	0.2	9	60	219
C	42	0.7	5	60	100
C	43	0.1		60	64
C	44	0.5	4	74	25
C	45	0.3	6		74
N2 Control	1	1	11	46	270
N2 Control	2	1	23	48	272
N2 Control	3	1	22	50	170
N2 Control	4	1	22	52	326
N2 Control	5	1	22	48	300
N2 Control	6	1	2		
N2 Control	7	1	14	48	305
N2 Control	8	1	15	48	233
N2 Control	9	1	22	46	332
N2 Control	10	1	19	48	276
N2 Control	11	1	16	46	347
N2 Control	12	1	22	46	283
N2 Control	13	1	23	46	291
N2 Control	14	1	14	48	291
N2 Control	15	1	11	46	311
N2 Control	16	0.9	5	48	286
N2 Control	17	1	6	44	347
N2 Control	18	1	17	42	314
N2 Control	19	1	14	48	347
N2 Control	20	1	22	50	319
N2 Control	21	1	14	48	201
N2 Control	22	1	14	48	187
N2 Control	23	1	18	52	285
N2 Control	24	1	10	50	347
N2 Control	25	1	22	46	312
N2 Control	26	1	12	50	313
N2 Control	27	1	20	50	330
N2 Control	28	1	10	48	321
N2 Control	29	1	10	48	316
N2 Control	30	1	18	48	356
N2 Control	31	1	18	48	292
N2 Control	32	1	18	42	283
N2 Control	33	1	2		

N2 Control	34	1	19	48	253
N2 Control	35	1	22	46	292
N2 Control	36	1	12	48	317
N2 Control	37	1	10	48	323
N2 Control	38	1	9	44	299
N2 Control	39	0.8	20	48	289
N2 Control	40	1	11	48	251
N2 Control	41	1	11	50	297
N2 Control	42	1	2		
N2 Control	43	1	22	48	379
N2 Control	44	1	17	48	299
N2 Control	45	1	9	48	295
N2 Control	46	1	10	48	358
N2 Control	47	1	10	48	327
N2 Control	48	0.9	25	48	327
N2 Control	49	1	18	48	303
N2 Control	50	1	23	46	344
N2 Control	51	1	20	48	322
N2 Control	52	0.9	2		
N2 Control	53	1	22	48	331
N2 Control	54	1	14	50	353
N2 Control	55	1	20	42	349
N2 Control	56	1	23	48	276
N2 Control	57	1	14	44	334
N2 Control	58	1	10	48	298
N2 Control	59	1	12	42	355
N2 Control	60	1	23	50	330
N2 Control	61	0.9	22	50	306
N2 Control	62	1	14	48	333
N2 Control	63	1	12	44	329
N2 Control	64	1	23	50	324
N2 Control	65	0.9	10	50	369
N2 Control	66	0.9	4	42	19
N2 Control	67	1	12	48	354
N2 Control	68	1	18	48	319
N2 Control	69	1	10	48	418
N2 Control	70	1	14	46	362
N2 Control	71	1	19	50	348
N2 Control	72	1	10	48	251
N2 Control	73	1	14	48	281
N2 Control	74	1	12		
N2 Control	75	1	14	44	333
N2 Control	76	1	8	48	304

N2 Control	77	1	13	50	333
N2 Control	78	1	20	48	366
N2 Control	79	1	8	50	376
N2 Control	80	1	10	50	307

Supplementary Table S2. Relative mtDNA copy-number was measured by ddPCR on genomic DNA from single worms. The intra-individual frequency of the *meuDf1* mitotype was estimated using a probe to (i) *nd1* and (ii) the deleted region of *nd4* in *meuDf1*, which yielded the concentration of total mtDNA and mtDNA with intact *nd4*, respectively. The frequency of mtDNA carrying the WT (intact) *nd4* was calculated from the following ratio: [concentration of the *nd4* deleted region/concentration of *nd1*]. The frequency of the *meuDf1* mitotype was calculated as 1-[concentration of the *nd4* deleted region/concentration of *nd1*].

**Sample description 1**	**Sample description 2**	**Sample description 3**	**Target**	**Conc. (copies/µL)**	**Experiment**	**Dye Name(s**	**Accepted Droplets**	**Positives**	**Negatives**	***meuDf1* frequency**
A1	Replay Parent		ND4	2,709.52	CNV	FAM	11826	10644	1182	0.315
A1	Replay Parent		ND1	3,958.05	CNV	HEX	11826	11417	409	
A1.1.1	Replay Line 1	Generation 1	ND4	1,758.97	CNV	FAM	13393	10390	3003	0.262
A1.1.1	Replay Line 1	Generation 1	ND1	2,384.21	CNV	HEX	13393	11628	1765	
A1.2.1	Replay Line 2	Generation 1	ND4	1,496.54	CNV	FAM	13420	9659	3761	0.251
A1.2.1	Replay Line 2	Generation 1	ND1	1,998.86	CNV	HEX	13420	10966	2454	
A1.3.1	Replay Line 3	Generation 1	ND4	772.81	CNV	FAM	12917	6220	6697	0.431
A1.3.1	Replay Line 3	Generation 1	ND1	1,357.55	CNV	HEX	12917	8843	4074	
A1.4.1	Replay Line 4	Generation 1	ND4	882.45	CNV	FAM	13967	7370	6597	0.456
A1.4.1	Replay Line 4	Generation 1	ND1	1,621.45	CNV	HEX	13967	10447	3520	
A1.5.1	Replay Line 5	Generation 1	ND4	1,146.58	CNV	FAM	14223	8856	5367	0.354
A1.5.1	Replay Line 5	Generation 1	ND1	1,773.85	CNV	HEX	14223	11074	3149	
A1.6.1	Replay Line 6	Generation 1	ND4	692.14	CNV	FAM	16259	7231	9028	0.284
A1.6.1	Replay Line 6	Generation 1	ND1	967.33	CNV	HEX	16259	9114	7145	
A1.7.1	Replay Line 7	Generation 1	ND4	1,175.30	CNV	FAM	15715	9928	5787	0.479
A1.7.1	Replay Line 7	Generation 1	ND1	2,256.24	CNV	HEX	15715	13406	2309	
A1.8.1	Replay Line 8	Generation 1	ND4	548.71	CNV	FAM	14809	5520	9289	0.382
A1.8.1	Replay Line 8	Generation 1	ND1	887.46	CNV	HEX	14809	7844	6965	
A1.9.1	Replay Line 9	Generation 1	ND4	932.81	CNV	FAM	12609	6903	5706	0.399
A1.9.1	Replay Line 9	Generation 1	ND1	1,552.35	CNV	HEX	12609	9239	3370	
A1.10.1	Replay Line 10	Generation 1	ND4	685.34	CNV	FAM	11132	4915	6217	0.218
A1.10.1	Replay Line 10	Generation 1	ND1	876.19	CNV	HEX	11132	5846	5286	
A1.11.1	Replay Line 11	Generation 1	ND4	1,474.70	CNV	FAM	9555	6827	2728	0.260
A1.11.1	Replay Line 11	Generation 1	ND1	1,992.30	CNV	HEX	9555	7798	1757	
A1.12.1	Replay Line 12	Generation 1	ND4	1,403.50	CNV	FAM	9884	6886	2998	0.340
A1.12.1	Replay Line 12	Generation 1	ND1	2,126.91	CNV	HEX	9884	8263	1621	
A1.14.1	Replay Line 14	Generation 1	ND4	1,269.53	CNV	FAM	12383	8174	4209	0.350
A1.14.1	Replay Line 14	Generation 1	ND1	1,952.69	CNV	HEX	12383	10028	2355	

A1.1.5	Replay Line 1	Generation 5	ND4	422.01	CNV	FAM	12212	3681	8531	0.723
A1.1.5	Replay Line 1	Generation 5	ND1	1,523.47	CNV	HEX	12212	8867	3345	
A1.2.5	Replay Line 2	Generation 5	ND4	1,310.72	CNV	FAM	13001	8734	4267	0.441
A1.2.5	Replay Line 2	Generation 5	ND1	2,344.61	CNV	HEX	13001	11229	1772	
A1.3.5	Replay Line 3	Generation 5	ND4	942.21	CNV	FAM	13414	7392	6022	0.596
A1.3.5	Replay Line 3	Generation 5	ND1	2,333.27	CNV	HEX	13414	11568	1846	
A1.4.5	Replay Line 4	Generation 5	ND4	62.65	CNV	FAM	13769	714	13055	0.862
A1.4.5	Replay Line 4	Generation 5	ND1	454.72	CNV	HEX	13769	4414	9355	
A1.5.5	Replay Line 5	Generation 5	ND4	437.85	CNV	FAM	10516	3268	7248	0.708
A1.5.5	Replay Line 5	Generation 5	ND1	1,501.00	CNV	HEX	10516	7580	2936	
A1.6.5	Replay Line 6	Generation 5	ND4	517.40	CNV	FAM	12315	4382	7933	0.525
A1.6.5	Replay Line 6	Generation 5	ND1	1,089.99	CNV	HEX	12315	7439	4876	
A1.7.5	Replay Line 7	Generation 5	ND4	619.32	CNV	FAM	13702	5608	8094	0.759
A1.7.5	Replay Line 7	Generation 5	ND1	2,570.72	CNV	HEX	13702	12161	1541	
A1.8.5	Replay Line 8	Generation 5	ND4	749.33	CNV	FAM	12762	6012	6750	0.660
A1.8.5	Replay Line 8	Generation 5	ND1	2,204.75	CNV	HEX	12762	10803	1959	
A1.9.5	Replay Line 9	Generation 5	ND4	465.22	CNV	FAM	13251	4328	8923	0.748
A1.9.5	Replay Line 9	Generation 5	ND1	1,843.57	CNV	HEX	13251	10486	2765	
A1.10.5	Replay Line 10	Generation 5	ND4	1,246.81	CNV	FAM	14019	9161	4858	0.117
A1.10.5	Replay Line 10	Generation 5	ND1	1,412.45	CNV	HEX	14019	9799	4220	
A1.11.5	Replay Line 11	Generation 5	ND4	1,190.57	CNV	FAM	12963	8251	4712	0.320
A1.11.5	Replay Line 11	Generation 5	ND1	1,751.13	CNV	HEX	12963	10037	2926	
A1.12.5	Replay Line 12	Generation 5	ND4	897.97	CNV	FAM	12462	6653	5809	0.721
A1.12.5	Replay Line 12	Generation 5	ND1	3,214.32	CNV	HEX	12462	11651	811	
A1.13.5	Replay Line 13	Generation 5	ND4	1,129.45	CNV	FAM	11753	7253	4500	0.527
A1.13.5	Replay Line 13	Generation 5	ND1	2,387.16	CNV	HEX	11753	10208	1545	
A1.14.5	Replay Line 14	Generation 5	ND4	1,095.83	CNV	FAM	12257	7428	4829	0.518
A1.14.5	Replay Line 14	Generation 5	ND1	2,274.62	CNV	HEX	12257	10484	1773	
A1.15.5	Replay Line 15	Generation 5	ND4	2,083.79	CNV	FAM	13878	11517	2361	0.107
A1.15.5	Replay Line 15	Generation 5	ND1	2,332.56	CNV	HEX	13878	11967	1911	
A1.1.10	Replay Line 1	Generation 10	ND4	106.66	CNV	FAM	12484	1082	11402	0.897
A1.1.10	Replay Line 1	Generation 10	ND1	1,037.37	CNV	HEX	12484	7315	5169	
A1.2.10	Replay Line 2	Generation 10	ND4	787.18	CNV	FAM	12041	5874	6167	0.717
A1.2.10	Replay Line 2	Generation 10	ND1	2,780.53	CNV	HEX	12041	10908	1133	
A1.3.10	Replay Line 3	Generation 10	ND4	337.64	CNV	FAM	13151	3281	9870	0.820
A1.3.10	Replay Line 3	Generation 10	ND1	1,874.47	CNV	HEX	13151	10478	2673	
A1.4.10	Replay Line 4	Generation 10	ND4	11.80	CNV	FAM	12224	122	12102	0.941
A1.4.10	Replay Line 4	Generation 10	ND1	200.22	CNV	HEX	12224	1913	10311	

A1.5.10	Replay Line 5	Generation 10	ND4	196.58	CNV	FAM	12081	1859	10222	0.856
A1.5.10	Replay Line 5	Generation 10	ND1	1,368.82	CNV	HEX	12081	8307	3774	
A1.6.10	Replay Line 6	Generation 10	ND4	100.25	CNV	FAM	12879	1052	11827	0.922
A1.6.10	Replay Line 6	Generation 10	ND1	1,289.75	CNV	HEX	12879	8576	4303	
A1.7.10	Replay Line 7	Generation 10	ND4	95.13	CNV	FAM	11960	929	11031	0.934
A1.7.10	Replay Line 7	Generation 10	ND1	1,437.61	CNV	HEX	11960	8436	3524	
A1.8.10	Replay Line 8	Generation 10	ND4	244.57	CNV	FAM	10831	2033	8798	0.847
A1.8.10	Replay Line 8	Generation 10	ND1	1,596.15	CNV	HEX	10831	8042	2789	
A1.9.10	Replay Line 9	Generation 10	ND4	166.66	CNV	FAM	10978	1450	9528	0.790
A1.9.10	Replay Line 9	Generation 10	ND1	793.81	CNV	HEX	10978	5387	5591	
A1.10.10	Replay Line 10	Generation 10	ND4	299.9	CNV	FAM	13079	2943	10136	0.725
A1.10.10	Replay Line 10	Generation 10	ND1	1,090.10	CNV	HEX	13079	7901	5178	
A1.11.10	Replay Line 11	Generation 10	ND4	372.08	CNV	FAM	12514	3393	9121	0.603
A1.11.10	Replay Line 11	Generation 10	ND1	937.6	CNV	HEX	12514	6874	5640	
A1.12.10	Replay Line 12	Generation 10	ND4	15.91	CNV	FAM	12653	170	12483	0.877
A1.12.10	Replay Line 12	Generation 10	ND1	129.62	CNV	HEX	12653	1320	11333	
A1.13.10	Replay Line 13	Generation 10	ND4	411.42	CNV	FAM	16045	4735	11310	0.685
A1.13.10	Replay Line 13	Generation 10	ND1	1,308.06	CNV	HEX	16045	10767	5278	
A1.14.10	Replay Line 14	Generation 10	ND4	395.06	CNV	FAM	16071	4584	11487	0.632
A1.14.10	Replay Line 14	Generation 10	ND1	1,072.58	CNV	HEX	16071	9613	6458	
A1.15.10	Replay Line 15	Generation 10	ND4	876.95	CNV	FAM	15771	8287	7484	0.308
A1.15.10	Replay Line 15	Generation 10	ND1	1,268.12	CNV	HEX	15771	10404	5367	

Supplementary Table S3. Relative mtDNA copy-number was measured by ddPCR. The relative mtDNA copy-number was estimated using a probe to a single-copy nuclear gene, *daf-1*, and the mitochondrial *nd1* gene.

**Relative mtDNA copy-number**	
***meuDf1*-bearing lines**	**N2 control bearing wildtype mtDNA**
63.9740	46.0374
224.8727	42.6530
338.7586	32.5160
177.8847	53.8826
51.2949	74.0156
58.6107	38.5422
346.4588	46.2815
30.0907	46.3316
57.1634	43.4716
71.5187	64.7132
98.9344	32.5165
82.3496	49.3284
63.7571	59.4270
57.4130	161.9490
46.8461	45.0399
56.2257	55.7804
74.3730	46.2815
61.5363	–
40.4493	–
122.0950	–
66.7637	–
65.5991	–
56.0661	–
49.7836	–
54.7491	–
52.6743	–
61.9562	–
60.3037	–
41.8762	–
61.2494	–
